# Perspectives of people living with HIV‐1 on implementation of long‐acting cabotegravir plus rilpivirine in US healthcare settings: results from the CUSTOMIZE hybrid III implementation‐effectiveness study

**DOI:** 10.1002/jia2.26006

**Published:** 2022-09-12

**Authors:** Cindy P. Garris, Maggie Czarnogorski, Marybeth Dalessandro, Ronald D'Amico, Toyin Nwafor, Will Williams, Deanna Merrill, YuanYuan Wang, Larissa Stassek, Michael B. Wohlfeiler, Gary I. Sinclair, Leandro A. Mena, Blair Thedinger, Jason A. Flamm, Paul Benson, William R. Spreen

**Affiliations:** ^1^ ViiV Healthcare Durham North Carolina USA; ^2^ ViiV Healthcare Collegeville Pennsylvania USA; ^3^ GlaxoSmithKline Collegeville Pennsylvania USA; ^4^ Evidera Bethesda Maryland USA; ^5^ AIDS Healthcare Foundation Los Angeles California USA; ^6^ Prism Health North Texas Dallas Texas USA; ^7^ University of Mississippi Medical Center Jackson Mississippi USA; ^8^ KC CARE Health Center Kansas City Missouri USA; ^9^ Kaiser Permanente Sacramento Sacramento California USA; ^10^ Be Well Medical Center Berkley California USA

**Keywords:** antiretroviral therapy, integrase strand transfer inhibitor, non‐nucleoside reverse transcriptase inhibitor, acceptability, appropriateness, treatment satisfaction

## Abstract

**Introduction:**

The CUSTOMIZE hybrid III implementation‐effectiveness study evaluated implementation of once‐monthly long‐acting (LA) cabotegravir + rilpivirine in diverse US healthcare settings. Here, we report patient participant perspectives after 12 months in CUSTOMIZE.

**Methods:**

CUSTOMIZE was a phase IIIb, 12‐month study conducted from July 2019 to October 2020 at eight diverse US HIV clinics that enrolled virologically suppressed people living with HIV‐1 (PLHIV) on a stable oral regimen to receive monthly cabotegravir + rilpivirine LA injections after a 1‐month oral lead‐in. Participants were administered quantitative surveys before injections at months 1 (baseline), 4 and 12. A randomly selected subset of participants was interviewed at baseline and month 12. Data collection at month 12 was completed by October 2020 (during the COVID‐19 pandemic).

**Results:**

At baseline, 109 and 34 participants completed surveys and interviews, respectively; 87% were male; 35% were Black or African American. All participants who remained in the study at month 12 (*n* = 102) maintained HIV‐1 RNA <50 copies/ml; two participants withdrew due to injection‐related reasons. Mean total scores measuring acceptability and appropriateness of cabotegravir + rilpivirine LA were high at baseline (4.5–4.6 out of 5) and month 12 (4.7–4.9). At month 12, 74% of participants reported nothing interfered with receiving LA injections; injection pain or soreness was the most common concern (15%). Time spent in the clinic and coming to the clinic for monthly injections was very or extremely acceptable after 12 months for most participants (93% and 87%, respectively), with 64% reporting having spent ≤30 minutes in the clinic for injection visits. At month 12, 92% of participants preferred LA injections to daily oral tablets (3%); 97% plan to continue LA treatment going forward. In month 12 interviews, 24 (77%) of 31 participants reported the COVID‐19 pandemic did not impact their ability to receive treatment.

**Conclusions:**

Once‐monthly cabotegravir + rilpivirine LA was highly acceptable among PLHIV who were virologically suppressed on a stable antiretroviral regimen and interested in trying LA therapy, with few participants reporting challenges receiving LA injections. Implementation data from CUSTOMIZE suggest that monthly LA injections provide a convenient and appealing treatment option for PLHIV.

## 1 INTRODUCTION

Cabotegravir + rilpivirine is the first complete long‐acting (LA) injectable regimen recommended by treatment guidelines and indicated for the maintenance of virologic suppression in people living with HIV‐1 (PLHIV) on a stable antiretroviral regimen [1, 2]. In the ATLAS and FLAIR phase III studies, 48 weeks of cabotegravir + rilpivirine LA administered every 4 weeks demonstrated non‐inferior efficacy to daily oral antiretroviral therapy (ART) in virologically suppressed adults with HIV‐1 infection [3, 4]. Cabotegravir + rilpivirine LA remained non‐inferior to daily oral ART through 96 weeks in FLAIR [5]. Administration of cabotegravir + rilpivirine LA every 8 weeks also demonstrated non‐inferiority to every 4‐week dosing at 48 and 96 weeks in the phase IIIb ATLAS‐2M study [6, 7].

Cabotegravir + rilpivirine LA administered once monthly or every 2 months provides PLHIV a less frequent dosing alternative to daily oral ART. The challenges some PLHIV experience with daily oral dosing, such as pill fatigue, remembering to take medications and concerns about stigma, may be addressed by LA therapy [8].

CUSTOMIZE is a hybrid III implementation‐effectiveness study evaluating healthcare provider and patient participant perspectives on the implementation of once‐monthly cabotegravir + rilpivirine LA in diverse US healthcare settings. Here, we report patient participant perspectives after 12 months of cabotegravir + rilpivirine LA implementation in CUSTOMIZE.

## 2 METHODS

### 2.1 Study design

CUSTOMIZE is a phase IIIb, 12‐month, multicentre, hybrid III implementation‐effectiveness study evaluating the acceptability, appropriateness and feasibility of cabotegravir + rilpivirine LA implementation at eight clinical sites in the United States (ClinicalTrials.gov identifier: NCT04001803). Patient participants received an oral lead‐in of cabotegravir 30 mg + rilpivirine 25 mg once daily for 1 month, a loading dose of cabotegravir 600 mg + rilpivirine 900 mg LA at month 1 (baseline), and monthly cabotegravir 400 mg + rilpivirine 600 mg LA maintenance injections thereafter. Injections were administered by clinic staff with a ±7‐day dosing window around the target visit date. Participants were provided with a toolkit of optional educational materials about the regimen in digital and hard copy formats, support items (i.e. hot and cold packs), an informational application and appointment reminder systems.

Participants were administered quantitative surveys at baseline, month 4 and month 12 before the first, fourth and 12th injections, respectively. A subset of participants was randomly selected to participate in qualitative telephone interviews at baseline and month 12. Baseline interviews were conducted between the start of the oral lead‐in period and the first injection visit and lasted ∼90 minutes. Month 12 interviews were conducted between the month 11 and 12 visits and lasted ∼60 minutes. Block randomization was used to select approximately four participants to be interviewed from each site. Because planned enrolment numbers were not achieved at every site, the last six participants enrolled in the study were automatically added to the interview group to ensure interviews were conducted in >30% of participants.

This study was conducted in accordance with the ethical principles outlined in the Declaration of Helsinki. The study protocol was approved by a central Institutional Review Board and/or local Institutional Review Boards where required. All participants provided written informed consent before enrolment. Participants received $25 as compensation for completing surveys and interviews.

### 2.2 Participants

Eligible participants were adults aged ≥18 years on a stable two‐ or three‐drug ART regimen for ≥6 months and with documented plasma HIV‐1 RNA <50 copies/ml at screening and within the previous 6 and 12 months before screening.

### 2.3 Endpoints and assessments

Proctor Framework was used to evaluate implementation outcomes, focusing on acceptability and appropriateness [9]. Implementation outcomes were assessed through quantitative survey questions using the previously validated acceptability of intervention measure (AIM) and intervention appropriateness measure (IAM) as well as bespoke questions, which were validated with cognitive testing [10]. The determinant framework used to guide semi‐structured qualitative interviews was the Consolidated Framework for Implementation Research (CFIR), which is composed of five domains: inner setting, outer setting, process, individual characteristics and intervention characteristics [11].

The primary quantitative endpoints were acceptability (AIM) and appropriateness (IAM) at baseline, month 4 and month 12. The AIM and IAM surveys have four items, which are rated on a scale of 1 (completely disagree) to 5 (completely agree) [10]. Raw AIM and IAM total scores were calculated by adding scores for each of the four items (range of 4–20) and then linearly transformed to a 1 to 5 scale, where 1 and 5 indicate the least and most acceptability or appropriateness, respectively. Items in the AIM survey were as follows: (1) cabotegravir + rilpivirine LA injection treatment meets my needs for treating my HIV, (2) cabotegravir + rilpivirine LA injection treatment is appealing to me, (3) I like cabotegravir + rilpivirine LA injections for treating my HIV and (4) I welcome cabotegravir + rilpivirine LA injections for treating my HIV. Items in the IAM survey were as follows: (1) cabotegravir + rilpivirine LA seems fitting for my life, (2) cabotegravir + rilpivirine LA seems suitable for my life, (3) cabotegravir + rilpivirine LA seems applicable to my life and (4) cabotegravir + rilpivirine LA seems like a good match for my life.

Additional survey items evaluated treatment preference, participant satisfaction with the process, and facilitators of and barriers to implementation. Treatment satisfaction was assessed using the 12‐item HIV Treatment Satisfaction Questionnaire, status version (HIVTSQs), with a rating scale for each item of 0 (very dissatisfied) to 6 (very satisfied). The CFIR framework was used in interviews to evaluate barriers to and facilitators of receiving injections as well as other factors affecting participants’ experiences with and view of cabotegravir + rilpivirine LA. Surveys and interviews conducted at baseline (i.e. before receiving cabotegravir + rilpivirine LA) evaluated participants’ initial impressions, expectations and perceptions of cabotegravir + rilpivirine LA.

Clinical outcomes included the proportion of participants with HIV‐1 RNA <50 copies/ml or confirmed virologic failure (CVF; two consecutive HIV‐1 RNA measurements ≥200 copies/ml), incidence and severity of adverse events (AEs) and injection site reactions (ISRs).

### 2.4 Data analyses

CUSTOMIZE utilized a “shotgun” enrolment strategy, with a pre‐defined enrolment period of 10 weeks. Enrolment of 135 participants (*n* = 15 per site) was planned for estimations of the primary endpoint; 115 participants were enrolled at eight sites (five sites met enrolment target), with one additional FQHC site withdrawing from the study before enrolling any participants due to staffing difficulties. Other sites that did not meet enrolment targets had experienced a delay in Institutional Review Board approval, resulting in only 10 available business days to enrol participants. The discrepancy between planned and enrolled participants did not substantively affect the analysis. Data collection for participant surveys and interviews ended by September 2019, January 2020 and October 2020 for baseline, month 4 (before COVID‐19 was detected in the United States) and month 12 (during the COVID‐19 pandemic), respectively. Demographic characteristics and survey data were summarized using descriptive statistics. Audio recordings of interviews were transcribed, systematically coded and thematically analysed using ATLAS.ti software (version 8.1; ATLAS.ti Scientific Software Development GmbH, Berlin, Germany). Not all participants were asked every interview question, and participants could provide ≥1 response to the same question; reported totals for interview data reflect the number of participants responding to a particular discussion topic out of the total number interviewed at baseline or month 12. Virologic outcomes were evaluated using the FDA Snapshot algorithm. Missing data for COVID‐19–related reasons were imputed using the last observation carried forward approach.

## 3 RESULTS

### 3.1 Study population

Of 146 participants screened, 115 enrolled in the study. Demographics were generally consistent between participants who were screened versus those who ultimately enrolled (male, 76% vs. 86%; White, 52% vs. 57%; Black or African American, 42% vs. 37%; mean age, 40 vs. 39 years). Data on the number of participants approached to participate in CUSTOMIZE were not recorded as part of the enrolment process. Overall, 109 completed surveys and received injections at baseline; of these, 34 participants also completed baseline interviews (Table S1). Participants completing surveys were from eight geographically diverse US clinics, with 34% from the Southeastern United States. Of participants completing surveys, most were male (87%) and White (59%) or Black or African American (35%); 27% were Hispanic or Latino; the mean age was 39 years (Table 1). Baseline demographics were generally comparable between the total sample of participants completing surveys and the subset completing interviews. At month 4, 106 participants completed surveys; at month 12, 102 and 31 participants completed surveys and interviews, respectively. In March 2020, when CUSTOMIZE was approximately halfway completed, much of the United States began COVID‐19 pandemic–related closures. By month 12 (late summer/early fall 2020), 19 (19%) of 102 participants had ≥1 COVID‐19–impacted visits, consisting of missed or rescheduled injection visits, quarantine, COVID‐19 diagnosis or clinic closure; none of these individuals withdrew from the study, and eight received short‐term oral cabotegravir + rilpivirine to cover missed injection visits. Among participants who received ≥1 cabotegravir + rilpivirine LA injection (*n* = 109), two withdrew from the study before month 12 for injection‐related reasons. Reasons for withdrawal among the other five participants who withdrew after receiving ≥1 injection included AEs (*n* = 3) and participant withdrawal (*n* = 2; due to out‐of‐state travel and participant relocation). Reasons for withdrawal among the six participants who withdrew during the oral lead‐in phase were protocol deviation (*n* = 3), physician decision based on exclusion criteria (*n* = 1), AEs (*n* = 1; diarrhoea, nausea, insomnia and mental disorder), and withdrawn consent due to the burden of procedures and frequency of injections and visits (*n* = 1).

**Table 1:** Baseline demographics

Parameter	Enrolled participants (*N* = 115)	Survey participants (*N* = 109)^a^	Interview participants (*N* = 34)
Age, mean (range), year	39 (20–65)	39 (20–65)	NA
20–29, *n* (%)	25 (22)	24 (22)	6 (18)
30–39, *n* (%)	45 (39)	42 (39)	18 (53)
40–49, *n* (%)	18 (16)	17 (16)	3 (9)
≥50, *n* (%)	27 (23)	26 (24)	7 (21)
Male, *n* (%)	99 (86)	95 (87)	28 (82)
Race and ethnicity, *n* (%)			
White or Caucasian or European heritage	66 (57)	64 (59)	18 (53)
Black or African American	42 (37)	38 (35)	14 (41)
American Indian or Alaskan Native	5 (4)	5 (5)	2 (6)
Multiple	2 (2)	2 (2)	0 (0)
Hispanic or Latino	30 (26)	29 (27)	9 (26)
Body mass index, median (range), kg/m^2^	27 (17–55)	27 (17–54)	NA

Abbreviation: NA, not available.

^a^ Participants receiving ≥1 cabotegravir + rilpivirine LA injection.

### 3.2 Clinical outcomes

**Figure 1:**
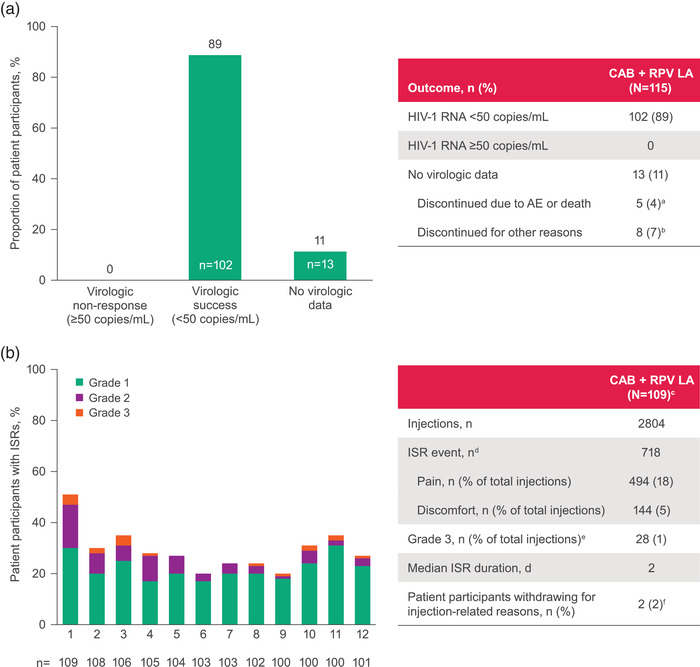
(a) Virologic outcomes at month 12 (modified Snapshot analysis with COVID‐19–related last observation carried forward) and (b) ISRs through month 12. Abbreviations: AE, adverse event; CAB, cabotegravir; ISR, injection site reaction; LA, long‐acting; RPV, rilpivirine. ^a^One death was reported due to diabetic ketoacidosis and drug abuse (both unrelated to study treatment). ^b^Reasons include withdrawn consent (*n* = 4), protocol deviation (*n* = 3) and physician decision (*n* = 1). ^c^Patient participants receiving ≥1 injection. ^d^Common ISRs reported in >1% of total injections. ^e^No grade 4 or 5 ISRs were reported. ^f^Injection site pain (*n* = 1) and injection site pain and injection intolerability (*n* = 1).

All 102 (89%) participants who completed through month 12 maintained HIV‐1 RNA <50 copies/ml at the month 12 visit (Figure 1a), and no confirmed virologic failures occurred. Through month 12, 104 (90%) of 115 patient participants experienced AEs, and 17 (15%) of those experienced grade ≥3 AEs; no grade 4 or 5 drug‐related AEs were reported (Table 2). The most common AE overall was ISRs, reported in 718 of 2804 total injections (Figure 1b and Table 2). Most (96%) ISRs were grade 1 or 2 and decreased in incidence after the first injection. Excluding ISRs, arthralgia (14%) and diarrhoea (14%) were the most common AEs (Table 2). Two patient participants withdrew for injection‐related reasons.

**Table 2:** Summary of adverse events, including injection site reactions, through month 12

Event, *n* (%)	CAB + RPV LA (*N* = 115)
Any AE	104 (90)
ISR events	78/109 (72)^a^
Grade ≥3 AE	17 (15)
Drug‐related AE	68 (59)
Grade ≥3 drug‐related AE^b^	3 (3)^c^
Serious AE	5 (4)^d^
Drug‐related serious AE	1 (1)^e^
AE leading to withdrawal	6 (5)^f^
Very common AEs (≥10%), excluding ISRs	
Arthralgia	16 (14)
Diarrhoea	16 (14)
Fatigue	14 (12)
Headache	13 (11)
Nausea	11 (10)
Insomnia	11 (10)

Abbreviations: AE, adverse event; CAB, cabotegravir; ISR, injection site reaction; LA, long‐acting; RPV, rilpivirine.

^a^ One hundred and nine participants received ≥1 injection.

^b^ No grade 4 or 5 drug‐related AEs were reported.

^c^ Injection site pain (*n* = 2) and mental status changes (*n* = 1).

^d^ Five participants reported 11 non‐ISR serious AEs.

^e^ Mental status changes.

^f^ Six participants reported 14 events leading to withdrawal: injection site pain (*n* = 2); arthralgia, back pain, diabetic ketoacidosis, diarrhoea, drug abuse, insomnia, lipodystrophy, mental disorder, myalgia, nausea, psoriasis and tendon pain (*n* = 1 each).

### 3.3 Implementation outcomes: proctor framework

#### 3.3.1 Acceptability and appropriateness

**Figure 2:**
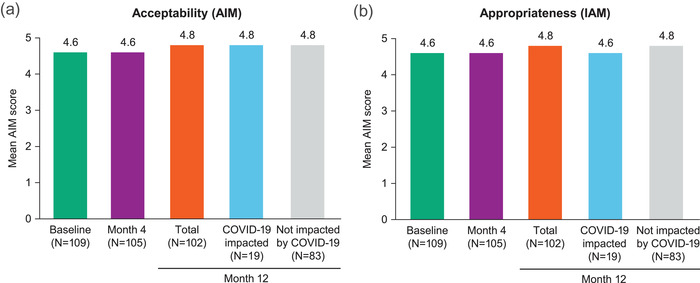
(a) Acceptability (AIM) and (b) appropriateness (IAM) of cabotegravir + rilpivirine LA injections over time and by COVID‐19 impact status at month 12. Both AIM and IAM were four‐item surveys that utilized a five‐point rating scale (1 = completely disagree to 5 = completely agree). Abbreviations: AIM, acceptability of intervention measure; CAB, cabotegravir; IAM, intervention appropriateness measure; LA, long‐acting; RPV, rilpivirine.

For acceptability, mean AIM scores at baseline and month 4 were high (4.6 out of 5) (Figure 2a). At month 12, during the COVID‐19 pandemic, the mean AIM score was 4.8 regardless of COVID‐19 impact status.

For the appropriateness of LA injections, mean IAM scores at baseline and month 4 were 4.6 out of 5 (Figure 2b). Mean total scores were 4.8 at month 12, with numerically higher scores observed for participants who were not impacted by COVID‐19 versus those who were impacted (4.8 vs. 4.6).

**Figure 3:**
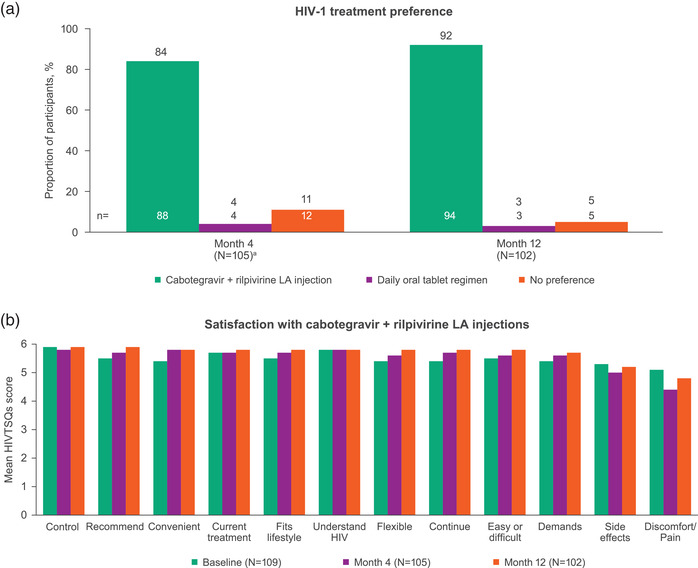
Participant‐reported (a) HIV‐1 treatment preference at months 4 and 12, and (b) level of satisfaction with cabotegravir + rilpivirine LA injections over time using the 12‐item HIVTSQs. The HIVTSQs items were rated on a scale of 0 to 6, where 0 = very dissatisfied and 6 = very satisfied. Abbreviations: HIVTSQs, HIV Treatment Satisfaction Questionnaire, status version; LA, long‐acting. ^a^One participant had missing data.

In month 4 surveys, 84% of participants preferred cabotegravir + rilpivirine LA compared with daily oral tablet regimens (4%), and 92% preferred LA injections by month 12 (vs. daily oral tablets [3%]; Figure 3a). The highest‐reported main advantages were frequency of administration (44%) and mode of administration (40%). Among 91 respondents, the main benefits of their preferred HIV‐1 therapy were that it was more convenient and easier to integrate into daily life (*n* = 61; 67%) and less stressful (*n* = 13; 14%).

In month 12 surveys, 99 (97%) participants reported they plan to continue using cabotegravir + rilpivirine LA for HIV‐1 treatment going forward, one (1%) planned to use their previous oral treatment and two (2%) planned to switch to another oral treatment.

#### 3.3.2 Treatment satisfaction

In baseline surveys, participant satisfaction with treatment was high, with an overall mean HIVTSQs score of 60.7 out of 66 points. Treatment satisfaction was numerically higher after 4 and 12 months of LA injections, with an overall mean (SD) changes from baseline in HIVTSQs scores of +1.50 (6.59) and +2.56 (6.18), respectively. At month 12, treatment satisfaction was high for each of the 12 HIVTSQs items (mean scores of 4.8–5.9 out of 6.0; Figure 3b).

#### 3.3.3 Feasibility

**Figure 4:**
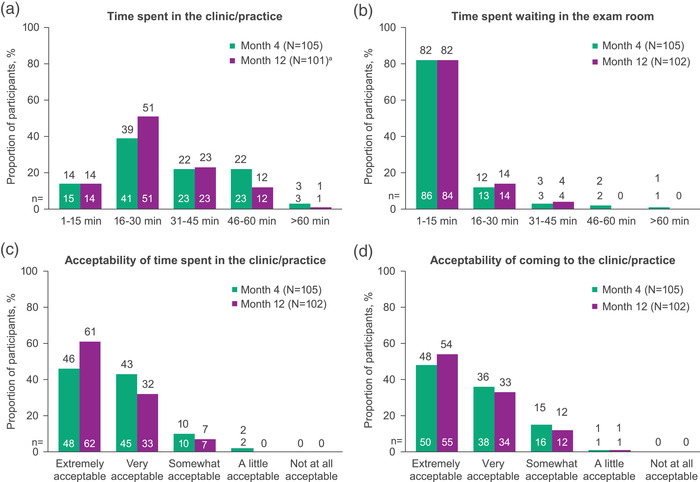
Participant‐reported (a) total time spent in the clinic/practice for each injection visit, (b) time spent in the exam room waiting for the injection, (c) acceptability of time spent in the clinic/practice for each injection visit and (d) acceptability of coming to the clinic/practice on a monthly basis for injection visits. ^a^One participant did not answer the question at month 12.

In month 12 surveys, more participants reported spending ≤30 minutes in the clinic for injection visits than at month 4 (64% vs. 53%; Figure 4a). Most (82%) participants reported spending 1–15 minutes in the exam room waiting for injections at months 4 and 12 (Figure 4b). Most participants reported time spent in the clinic for injection visits was very or extremely acceptable at month 4 (89%) and month 12 (93%; Figure 4c). At month 12, 87% of participants reported that coming to the clinic for monthly injection visits was very or extremely acceptable (Figure 4d).

**Figure 5:**
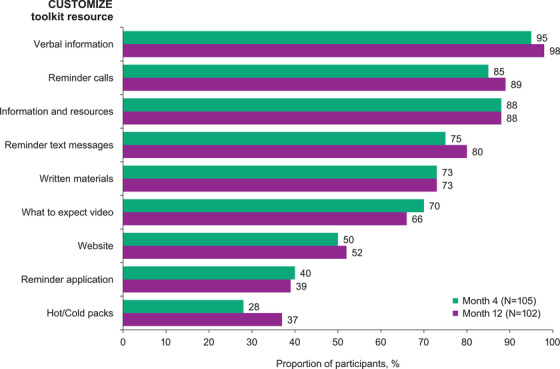
Participant‐reported perceived helpfulness of CUSTOMIZE toolkit resources at months 4 and 12. The bar graph indicates the proportion of participants who reported each resource as being very or extremely helpful in surveys.

Healthcare providers and participants were provided with a toolkit or resources intended to facilitate and support the feasibility of implementation. In surveys at months 4 and 12, participants indicated the most useful resources were verbal information relayed by their healthcare provider, appointment reminder calls and the clinical resources provided about the study and LA regimen, with ≥88% of participants reporting each resource was very or extremely helpful at month 12 (Figure 5). The resources rated as least helpful at months 4 and 12 were the mobile application appointment reminder (40% and 39%, respectively) and hot and cold packs (28% and 37%, respectively).

**Figure 6:**
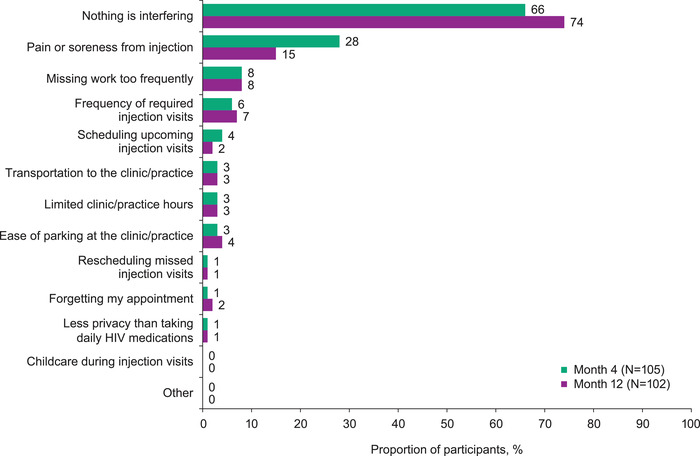
Participant‐reported barriers to receiving cabotegravir + rilpivirine LA injections at months 4 and 12. Participants were asked to select all options that applied from a prespecified list of 11 factors or barriers, an “other” response option and a “nothing is interfering with getting this treatment” response option. Abbreviation: LA, long‐acting.

Few participants reported barriers to receiving cabotegravir + rilpivirine LA injections, with the most common being injection pain or soreness (28% in month 4 and 15% in month 12 surveys; Figure 6). Other barriers were each identified by <10% of participants. Of four overlapping prespecified barrier response options between participant and healthcare staff surveys, a much lower proportion of participants than healthcare staff reported each option as a perceived barrier, including injection pain or soreness (15% and 48%, respectively; Figure S1).

### 3.4 CFIR determinant outcomes

#### 3.4.1 Individual characteristics: self‐efficacy and individual stage of change

In baseline interviews (*N* = 34), participant rationale for starting cabotegravir + rilpivirine LA was largely personal and logistical. Many participants indicated the increased convenience of monthly injections and removal of oral pills as a daily reminder of their HIV status was welcome in their lives. Altruism was also noted as a leading reason for switching to cabotegravir + rilpivirine LA. In particular, 16 (47%) participants indicated a desire to help other PLHIV or advance medical science by participating in CUSTOMIZE.

#### 3.4.2 Intervention characteristics: relative advantage and complexity

**Figure 7:**
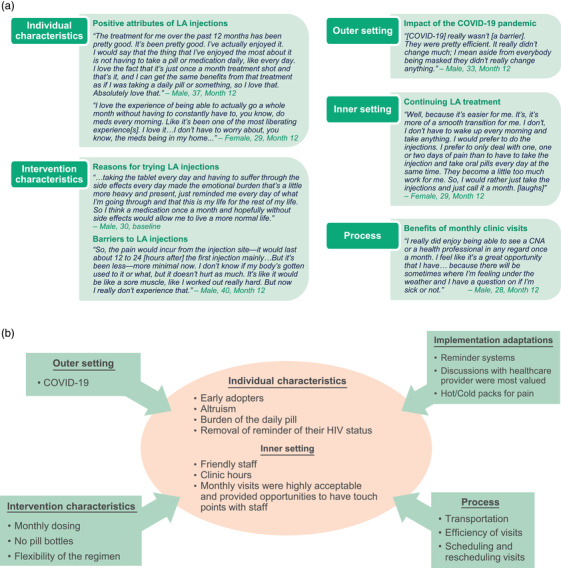
(a) Direct participant quotations from Consolidated Framework for Implementation Research–guided interviews at baseline and month 12, and (b) implementation results from the CUSTOMIZE study by Consolidated Framework for Implementation Research domain. Abbreviations: CNA, certified nursing assistant; LA, long‐acting.

In baseline interviews, participant‐reported reasons for wanting to switch to cabotegravir + rilpivirine LA included reduced dosing frequency (*n* = 13; 38%), potential for fewer side effects (*n* = 5; 15%) and reduced burden of daily oral medications (*n* = 4; 12%; Figure 7a). Seven (21%) participants felt that remembering to take daily pills resulted in an emotional burden. Many of these themes were reinforced in month 12 interviews (*N* = 31), with >50% of participants (*n* = 16) reporting they liked the convenience of LA injections compared with a daily oral pill. Other relative advantages included quick and easy clinic visits (*n* = 7; 23%), scheduling that fit their lifestyle (*n* = 7; 23%) and that the injections provided more privacy than pills (*n* = 6; 19%).

In month 12 interviews, participant‐reported dislikes about LA treatment included injection site pain (*n* = 21; 68%) and injection site swelling, lumps or knots and bruising (*n* = 17; 55%); two (6%) participants reported a fear of needles. Eight (26%) participants mentioned these challenges were experienced earlier in the study and improved over time (Figure 7a).

In month 12 interviews, 29 (94%) participants indicated they planned to continue cabotegravir + rilpivirine LA injections. The most common reasons for wanting to continue treatment included not wanting to go back to taking oral pills (*n* = 12; 39%) and convenience (*n* = 7; 23%; Figure 7a). Two (6%) participants indicated in interviews they did not plan to continue LA injections because they experienced a lot of injection site pain.

#### 3.4.3 Intervention characteristics: evidence strength and quality

In month 12 interviews, most participants (*n* = 22; 71%) reported they felt very informed about cabotegravir + rilpivirine LA injections; of those, 19 (86%) mentioned feeling informed because of information shared by a healthcare provider.

#### 3.4.4 Outer setting: external policy and incentives

In month 12 interviews, 24 (77%) of 31 participants reported the COVID‐19 pandemic did not impact their ability to receive treatment (Figure 7a). Six (19%) participants reported treatment was impacted by COVID‐19 for reasons, including needing to wear masks (*n* = 2) and childcare issues due to school closures, injection‐only clinic visits and separate clinic entrances (*n* = 1 each). At month 12, 18 (95%) participants with COVID‐19–impacted visits preferred LA injections (vs. daily oral therapy [*n* = 1; 5%]) and planned to continue LA therapy going forward.

#### 3.4.5 Outer setting: patient needs and resources

In baseline interviews, participants indicated transportation (*n* = 6; 18%), treatment costs (*n* = 3; 9%) and scheduling difficulties (*n* = 3; 9%) might be external barriers for other PLHIV to adopt LA injections; at month 12, 8 (26%) of 31 participants indicated transportation was a barrier for themselves. In month 12 surveys, <5% of participants reported each transportation‐related barrier for themselves, including transportation to the clinic and ease of clinic parking (Figure 6).

#### 3.4.6 Inner setting: structural characteristics

In month 12 interviews, 21 (68%) participants described positive aspects of coming to the clinic on a monthly basis, including liking to see familiar faces and feeling “at home” in the clinic (*n* = 13) and having the opportunity to ask other medical questions (*n* = 9).

#### 3.4.7 Process: planning, engaging and executing

In month 12 interviews, 10 participants (32%) described the form of transportation they used to get to their injection visits. Seven participants (23%) used a personal vehicle, two (6%) used public transit (bus and/or train), one (3%) took a cab and one (3%) used either Medicaid transport or borrowed a vehicle from their parents.

In month 12 interviews, many participants described injection visits as progressing like normal doctor's visits. Participants noted that blood work was required at alternating visits, occurring after their injection for some participants and at the start of the visit while their medication warmed to room temperature for others. Female participants of reproductive age noted needing to provide a urine sample for a pregnancy test.

In month 12 interviews, no participant complained about the length of injection visits, with 23% of participants describing being seen very quickly after check‐in (i.e. waiting <15 minutes). In month 12 surveys, 87% of participants reported spending ≤45 minutes in the clinic or practice for injection visits. Most participants in month 12 interviews reported not having issues coming to the clinic for monthly visits (*n* = 17; 55%; Figure 7a).

By month 12 interviews, the process of scheduling monthly appointments differed dramatically by individual and clinic type. Most participants (*n* = 25; 81%) reported that the “target date” approach with the ±7‐day dosing window was easy to understand. Most participants reported scheduling their appointments to occur on specific days based on their work schedule; one (3%) noted they sometimes scheduled appointments based on the availability of their preferred injector. Another participant (3%) noted they usually scheduled appointments for one of the last days of the month because that was when bus passes were issued. Most participants (*n* = 18; 58%) preferred morning appointments because it gave them time to manage the pain (i.e. it allowed them to be more active to reduce pain). Six participants (19%) preferred afternoon or evening appointments, usually because of work schedules, all of whom were from either private practices (*n* = 3; 43%) or federally qualified health centres (*n* = 3; 27%). At university sites and AIDS Healthcare Foundations, 100% (*n* = 5) and 75% (*n* = 3) of participants preferred morning appointments compared with 43–50% of participants from other clinic types.

#### 3.4.8 Process: reflecting and evaluating

Overall, participants reported few barriers to the implementation of cabotegravir + rilpivirine LA across all clinic types. Injection pain or soreness was reported as a barrier by 15% of participants in the month 12 surveys. Other logistical challenges, such as transportation, scheduling and rescheduling injection visits, and attending clinic visits during the COVID‐19 pandemic were negligible for participants. Minor adaptations to logistics led to the easy implementation and integration of cabotegravir + rilpivirine LA into routine care for the vast majority of participants.

## 4 DISCUSSION

Through 12 months of cabotegravir + rilpivirine LA implementation in CUSTOMIZE, PLHIV who were virologically suppressed on a stable regimen from diverse US healthcare settings found monthly cabotegravir + rilpivirine LA highly acceptable and appropriate for their lifestyles. Despite healthcare disruptions caused by the COVID‐19 pandemic, mean acceptability and appropriateness scores remained high at month 12, including for individuals who had COVID‐19–impacted injection visits. Cabotegravir + rilpivirine LA was highly effective among this cohort of cabotegravir‐naive participants, with no confirmed virologic failures occurring through 12 months of treatment. Results from this study suggest monthly cabotegravir + rilpivirine LA is an appealing treatment option for PLHIV who are interested in trying an LA regimen. Overall, the CFIR determinant outcomes reported in this analysis provide valuable insight into cabotegravir + rilpivirine LA implementation from the perspective of PLHIV (Figure 7b).

Participants reported few barriers to receiving LA therapy, with 74% of participants reporting no challenges receiving monthly injections at month 12. Although some participants indicated injection pain or soreness as a barrier, the proportion of participants reporting this concern decreased over time. Time spent in the clinic and coming to the clinic for monthly injection visits was highly acceptable after 12 months for most participants. Many participants described positive aspects associated with attending clinic visits, including increased opportunities to ask healthcare staff other medical questions. Therefore, the monthly clinic visits needed to receive monthly LA injections may provide additional benefits to PLHIV. Although the majority of participants in CUSTOMIZE reported no challenges receiving injections after 12 months, additional barriers will likely be present among individuals receiving cabotegravir + rilpivirine LA in the real world. For example, cabotegravir + rilpivirine LA may not be covered by a patient's health insurance or may require prior authorization, which limits or delays access for PLHIV interested in switching to an LA regimen [12].

Among this population of individuals in CUSTOMIZE interested in receiving LA therapy, cabotegravir + rilpivirine LA was highly preferred compared with daily oral ART (92% vs. 3%) after 12 months. Similar results were observed after 48 weeks in ATLAS and FLAIR, with a combined 523 (98%) of 532 responding participants preferring cabotegravir + rilpivirine LA [3, 4, 13]. At month 12 in CUSTOMIZE, 97% of participants planned to continue cabotegravir + rilpivirine LA moving forward, with individuals noting they liked the convenience and reduced stress associated with LA injections. Similarly, in the phase IIb LATTE‐2 study, ≥99% of participants who received cabotegravir + rilpivirine LA every 4 weeks (99 of 100) or every 8 weeks (107 of 108) reported they were highly satisfied to continue LA therapy after 96 weeks of treatment [14]. Overall, these results indicate that most virologically suppressed PLHIV on a stable regimen who choose LA therapy find the regimen satisfactory.

CUSTOMIZE enrolled a diverse participant population from a variety of clinic types and geographic locations. Participants were recruited from eight US clinics of five different types across seven states, with 34% of participants enrolling in four clinics in the Southeastern United States. Demographic characteristics of participants who were screened, enrolled, and completed surveys and interviews in CUSTOMIZE were generally representative of those for PLHIV in the United States, with 87% of participants completing surveys being male, 35% being Black or African American and 27% being Hispanic or Latino (vs. 77%, 41% and 23% in national data, respectively) [15]. Therefore, the study population was demographically similar to the overall population of PLHIV in the United States, increasing the likelihood that findings from this study can be generalized to a broader population.

This study has limitations. The overall sample size was relatively small as participant enrolment was limited by the study design; additional implementation concerns may emerge when more PLHIV receive LA ART at individual clinics. Of 115 enrolled participants, 13 (11%) withdrew from the study (*n* = 5 due to AEs), seven of whom withdrew after receiving cabotegravir + rilpivirine LA injections; these individuals may have prolonged detectable drug concentrations, which may increase the risk of drug resistance if viral suppression is not maintained on a subsequent oral regimen. Because CUSTOMIZE was conducted before regulatory approval of cabotegravir + rilpivirine LA in the United States, certain aspects of this study were not real world for participants, such as receiving free study medication and undergoing study‐specific procedures (i.e. more laboratory assessments). Many clinics were not operating normally during the COVID‐19 pandemic, providing an unanticipated real‐world challenge. Interviews were limited by time, so not all participants provided answers to every question. In this single‐arm study, PLHIV voluntarily and knowingly consented to receive LA treatment and were early adopters of LA ART, which may have influenced initial positive perceptions. It is assumed that PLHIV would also voluntarily start and continue LA treatment in the real world. However, an important finding is that these positive perspectives continued to increase over time despite implementation challenges, such as the COVID‐19 pandemic.

## 5 CONCLUSIONS

Through 12 months of implementation in diverse US healthcare settings, once‐monthly cabotegravir + rilpivirine LA was found to be highly acceptable and appropriate among PLHIV who were virologically suppressed on a stable regimen and interested in trying LA therapy, with few participants experiencing challenges receiving injections. Overall, implementation data from CUSTOMIZE demonstrate that monthly LA injections provide a convenient, appealing and effective treatment option that is preferred by many PLHIV over daily oral therapy.

## COMPETING INTERESTS

CPG, MC, MD, RD, TN, DM and WRS are employees of ViiV Healthcare and may own stock in GlaxoSmithKline. WW and YW are employees of and may own stock in GlaxoSmithKline. LS is an employee of Evidera, which receives funding from ViiV Healthcare for their work. MBW serves on the Janssen HIV Prophylactic Vaccine Advisory Board. GIS reports grants and personal fees from Janssen, ViiV Healthcare, and Merck and grants from Gilead Sciences. LAM reports grants and personal fees from Gilead Sciences, GlaxoSmithKline, ViiV Healthcare and MSD and grants from Janssen, Visby Medical, ThaiMed, Evofem Biosciences, SpeedDx Pty Ltd and Lupin Pharmaceutical outside of the submitted work. BT owns stock in ViiV Healthcare. JAF has served as a principal investigator for ViiV Healthcare. PB has nothing to disclose.

## AUTHORS’ CONTRIBUTIONS

MC, CPG, RD, LS and WRS contributed to the conception of the study. MC, CPG, MD, RD, TN, WW, DM, YW, LS and WRS contributed to the design of the study. WW, DM, LS, MBW, GIS, LAM, BT, JAF and PB contributed to the acquisition of data. MC, CPG, MD, RD, TN, YW and LS contributed to the analysis and interpretation of data. MC, CPG and LS contributed to drafting the manuscript. All authors contributed to critically revising the manuscript for important intellectual content and approve the manuscript for publication.

## Supporting information


**Figure S1**. Figure showing participant‐ and healthcare staff‐reported barriers to implementation among prespecified response options that overlapped between participant and healthcare staff surveys at month 12.

**Table S1**. Table showing participant enrolment.

## Data Availability

Anonymized individual participant data and study documents can be requested for further research from www.clinicalstudydatarequest.com.
