# Supplementary figures and images for: Perspectives of people living with HIV‐1 on implementation of long‐acting cabotegravir plus rilpivirine in US healthcare settings: results from the CUSTOMIZE hybrid III implementation‐effectiveness study
Source: J Int AIDS Soc. 2022 Sep 12;25(9):e26006. doi: 10.1002/jia2.26006 (PMC9468562; doi:10.1002/jia2.26006)

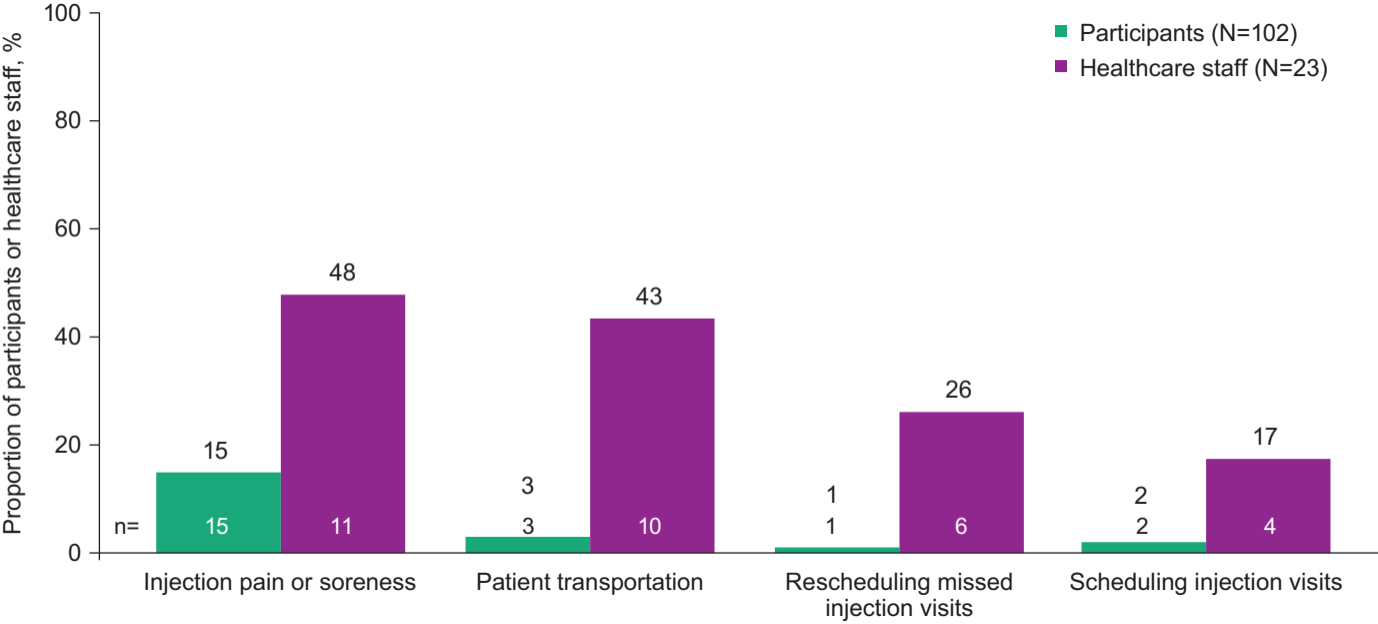

Supplement: Supplementary file 1 — Figure S1. Figure showing participant‐ and healthcare staff‐reported barriers to implementation among prespecified response options that overlapped between participant and healthcare staff surveys at month 12. [file JIA2-25-e26006-s002.pdf]
