# Supplementary material for: Perspectives of people living with HIV‐1 on implementation of long‐acting cabotegravir plus rilpivirine in US healthcare settings: results from the CUSTOMIZE hybrid III implementation‐effectiveness study
Source: J Int AIDS Soc. 2022 Sep 12;25(9):e26006. doi: 10.1002/jia2.26006 (PMC9468562; doi:10.1002/jia2.26006)
Supplement: Supplementary file 2 — Table S1. Table showing participant enrolment. [file JIA2-25-e26006-s001.docx]

| **Table S1. Participant Enrollment in CUSTOMIZE** | | | | | | |
| --- | --- | --- | --- | --- | --- | --- |
|  |  | **Baseline, n** | | **Month 4, n** | **Month 12, n** | |
| **Location** | **Clinic type** | **Surveys (N=109)** | **Interviews (N=34)** | **Surveys (N=106)^a^** | **Surveys (N=102)** | **Interviews (N=31)** |
| Atlanta, GA | Private practice | 9 | 3 | 8 | 8 | 3 |
| Detroit, MI | Private practice | 19 | 4 | 19 | 18 | 4 |
| Dallas, TX | FQHC | 18 | 6 | 18 | 17 | 6 |
| Kansas City, MO | FQHC | 19 | 5 | 19 | 18 | 5 |
| Jackson, MS | University | 8 | 3 | 7 | 7 | 2 |
| Jacksonville, FL | University | 5 | 3 | 5 | 5 | 3 |
| Miami, FL | AHF | 15 | 4 | 15 | 15 | 4 |
| Sacramento, CA | HMO | 16 | 6 | 15 | 14 | 4 |
| AHF, AIDS Healthcare Foundation; FQHC, federally qualified health center; HMO, health maintenance organization. a1 participant completed the Month 4 survey and withdrew from the study before receiving the Month 4 injection; 1 participant received the Month 4 injection but did not complete the Month 4 survey. | | | | | | |
